# Uniaxial Mechanical Behavior and Constitutive Modeling of Early-Age Steel Fiber-Reinforced Concrete Under Variable-Temperature Curing Conditions

**DOI:** 10.3390/ma18153642

**Published:** 2025-08-02

**Authors:** Yongkang Xu, Quanmin Xie, Hui Zhou, Yongsheng Jia, Zhibin Zheng, Chong Pan

**Affiliations:** 1 State Key Laboratory of Precision Blasting, Jianghan University, Wuhan 430056, China; xuyongkang0828@163.com (Y.X.); sinoblaster_jia@jhun.edu.cn (Y.J.); zzbblast@163.com (Z.Z.); pc20010610@163.com (C.P.); 2 Hubei Key Laboratory of Blasting Engineering, Jianghan University, Wuhan 430056, China

**Keywords:** high geothermal tunnels, early-age concrete, uniaxial compression, machine learning, constitutive model, fractal analysis

## Abstract

In high geothermal tunnels (>28 °C), curing temperature critically affects early-age concrete mechanics and durability. Uniaxial compression tests under six curing conditions, combined with CT scanning and machine learning-based crack analysis, were used to evaluate the impacts of curing age, temperature, and fiber content. The test results indicate that concrete exhibits optimal development of mechanical properties under ambient temperature conditions. Specifically, the elastic modulus increased by 33.85% with age in the room-temperature group (RT), by 23.35% in the fiber group (F), and decreased by 26.75% in the varying-temperature group (VT). A Weibull statistical damage-based constitutive model aligned strongly with the experimental data (R^2^ > 0.99). Fractal analysis of CT-derived cracks revealed clear fractal characteristics in the log(Nr)–log(r) curves, demonstrating internal damage mechanisms under different thermal histories.

## 1. Introduction

As China’s transportation infrastructure construction extends into the geologically complex mountainous regions of the west, the number of high geothermal tunnels has increased significantly, particularly in the mountainous and water-rich southwestern region of Sichuan Province. During tunnel blasting excavation, fracturing of the surrounding rock allows internal high-temperature groundwater to seep onto the newly excavated tunnel face. Measurements indicate that in some tunnel sections, the temperature of this seeping groundwater can exceed 92 °C. At this stage, the sprayed concrete used for primary tunnel lining is applied in direct contact with this high-temperature groundwater, exposing it to an environment characterized by both high temperature and high humidity. This exposure significantly impacts the early-age material properties of the sprayed concrete. Furthermore, the tunnel ventilation system rapidly cools the high-temperature groundwater, typically reducing it to ambient temperature within approximately 3 to 4 days. Consequently, the sprayed concrete in high geothermal tunnels is subjected to a time-varying thermo-hygrometric environment, inducing substantial alterations in its mechanical performance. This, in turn, poses a significant challenge to the long-term stability of tunnel support structures. As the core material of primary support, the time-dependent mechanical properties of early-age sprayed concrete directly affect tunnel construction safety and long-term structural durability [1,2,3]. Therefore, investigating the material properties, damage and failure modes, and constitutive models of early-age sprayed concrete under such high-temperature time-varying thermal environments is imperative for gaining a deeper understanding of its mechanical response within high geothermal tunnels.

Advancements in testing methods and measurement technologies have enabled the widespread use of X-ray computed tomography (CT) for investigating internal damage patterns in concrete materials after mechanical testing [4,5,6]. Ren et al. [7]’s CT scan analysis revealed that basalt fibers can significantly mitigate meso-structural deterioration in porous asphalt concrete under hydrodynamic loading, thereby enhancing its moisture damage resistance. Liang et al. [8] employed CT scanning and numerical simulation to reveal the multi-scale pore structure characteristics of aerated ceramsite concrete and their influence on the material’s failure mechanism, finding that cracks primarily initiate in high-porosity regions with lower strength. Wang et al. [9] integrated in situ 4D CT experimentation with four-phase meso-scale simulations to quantify the mechanical variability and fiber distribution patterns in high-toughness recycled aggregate concrete (HTRAC) incorporating copper-plated micro steel fibers, establishing a constitutive prediction model based on uniaxial compression tests. Li et al. [10] conducted accelerated acid-rain corrosion simulations and CT-based compressive testing, demonstrating that corrosion layers dominate the failure process, and revealing a negative linear relationship between pore area and compressive strength. Similarly, Xie et al. [11] used CT image analysis and *three-dimensional reconstruction technology* to examine concrete exposed to rapid freeze–thaw cycles, revealing the evolving damage of interfacial transition zones, and verifying the effectiveness of rapid-thaw laboratory tests. Despite the extensive use of CT technology in concrete research, challenges remain in accurately extracting and analyzing crack features from CT images—particularly in managing complex crack morphologies, high noise levels, and limited image resolution. There is an urgent need for intelligent algorithms or software to identify and extract cracks from CT slices. Chen et al. [12] achieved efficient and precise large-scale automatic counting of rice seedlings by combining high-resolution drone imagery with deep learning models, where YOLOv8n demonstrated optimal performance when trained on 200 images at a 12-m altitude, providing an innovative approach for rice phenotyping analysis. Huang et al. [13] proposed a rapid imaging and identification method for moving point radiation sources based on sequential Bayesian analysis, which enables fast identification of mobile radiation sources under extremely low signal-to-noise conditions by sequentially analyzing the spatiotemporal information of radiation particles. Nakamura et al. [14] utilized the open-source software “Fiji Image J” developed by the National Institutes of Health to measure the surface area ratio of specified elastin blue-stained regions, demonstrating that the ELST-blue score obtained through endoscopic ultrasound elastography can serve as a novel quantitative imaging biomarker for assessing endocrine dysfunction in early-stage chronic pancreatitis patients.

Additionally, developing robust damage constitutive models to describe the failure behavior of concrete remains a critical task [15,16]. Many researchers have developed such models under various loading conditions by integrating damage mechanics theories with statistical approaches [17,18,19]. For instance, Neuner et al. [20] developed and applied a series of concrete constitutive models, including an advanced damage–plasticity formulation, within 2D finite element simulations of NATM tunnel excavation to assess their impact on stress–displacement evolution in deep tunnel linings, with their predictive accuracy validated against in situ monitoring data. Constitutive models serve as a critical link between material behavior and engineering applications. Therefore, the development of models capable of accurately describing nonlinear deformation processes is essential.

High-temperature environments are known to accelerate cement hydration, induce early microcrack formation, and exacerbate moisture loss and fiber–matrix interface degradation, resulting in complex, multi-field coupled damage mechanisms [21,22]. However, most existing studies have focused on concrete behavior under standard-temperature curing conditions. The unique “spraying–heating–cooling” thermal histories typical of concrete in high geothermal tunnel construction—and their influence on early-age damage evolution—have not been systematically addressed.

This study focuses on the critical issue of early-age performance deterioration of concrete in high geothermal tunnels. Through a series of multifactor coupling experiments (including hydration age, temperature history, and fiber content), combined with CT imaging and machine learning-based crack recognition, we reveal the uniaxial compression damage evolution of concrete under variable temperature conditions. Furthermore, a statistical damage constitutive model incorporating temperature history effects is developed based on damage mechanics theory and Weibull distribution functions. This research goes beyond traditional macroscopic testing by integrating macro- and meso-scale analyses. The fractal dimensions of cracks in concrete are quantified using CT images, providing a new metric for durability assessment. This approach establishes a theoretical foundation for optimizing concrete mix design and construction curing practices in high geothermal tunnels.

## 2. Materials and Methods

### 2.1. Mix Proportion and Specimen Preparation

The mix proportion of C30 concrete used in this study was sourced from a high geothermal tunnel construction site. The material components are detailed in Table 1 and include the following:

Cement: P·O 42.5 ordinary Portland cement, supplied by Sichuan Yadong Cement Co., Ltd. Aggregates: Manufactured sand (particle size: 1–2 mm, fineness modulus: 2.8) and 5–10 mm continuously graded crushed stone (mainly composed of CaCO_3_), both obtained from the Songjiagou Self-Quarried Aggregate Plant. Admixtures: High-performance water reducer (water-reducing rate: 27.3%) and accelerator, provided by the Fushun Industrial Branch of CREC 11 Bureau Group Co., Ltd. (Wuhan, China). Fiber: Steel fibers produced by Liaocheng Hongshengyuan Metal Products Co., Ltd. (Liaocheng, China) (length: 1.5 mm, diameter: 0.3 mm, tensile strength: 600 MPa). Water: Ordinary surface water (tap water).

Laboratory testing employed cast-molding techniques. Based on scaled-down conversions from field data, 18 standard cylindrical specimens measuring 50 mm in diameter and 100 mm in height were prepared. These specimens were grouped by curing conditions and labeled as RT-1-24h to RT-3-24h, RT-4-72h to RT-6-72h, VT-1-24h to VT-3-24h, VT-4-72h to VT-6-72h, F-1-24h to F-3-24h, and F-4-72h to F-6-72h. Regarding the specimen naming convention (e.g., RT-1-24h): RT = curing condition (room temperature); 1 = specimen identification number; 24 h = curing duration (24 h). Additionally, six specimens were cast for each curing condition group (RT, VT, and F). Specimens numbered 1–3 underwent 24-h curing, while specimens 4–6 received 72-h curing.

Given that concrete in high-temperature tunnel environments is exposed to elevated temperatures immediately upon application to the surrounding rock, the laboratory specimens were placed in a high-temperature curing chamber immediately after casting. To ensure surface fitness and parallelism, the specimens were ground and polished after 24 h of curing. The end-face parallelism was controlled within 0.05 mm.

### 2.2. Testing Equipment and Experimental Design

**Figure 1:**
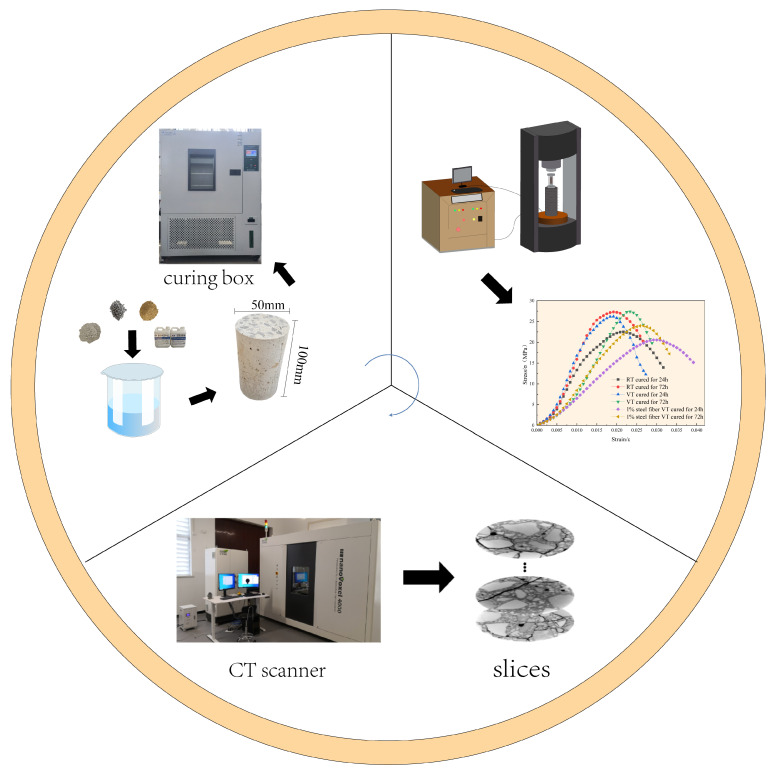
Experimental equipment and testing workflow.

The experimental setup and equipment are illustrated in Figure 1. The primary equipment included a temperature-controlled curing chamber, a triaxial loading meter, and a CT scanner. The curing chamber was a GT-TH-800F constant-temperature and -humidity environmental test chamber, capable of operating within −40 °C to 150 °C for both constant and variable temperature conditions. The three-axis loading device was an RIST-416 multi-field coupled material mechanics testing machine, capable of performing both uniaxial and triaxial loading tests. The CT device was a nanoVoxel-4000 open-tube reflective high-penetration CT system, equipped with a 3000 kV microfocus X-ray source, providing high-resolution internal imaging with strong penetration. The curing chamber was manufactured by Gaotian Test Equipment Co., Ltd. (Wuhan, Hubei Province, China); the uniaxial loading apparatus by Ruist Testing Instruments Co., Ltd. (Changchun, Jilin Province, China); and the CT scanning equipment by Sanying Precision Instruments Co., Ltd. (Tianjin, China).

**Figure 2:**
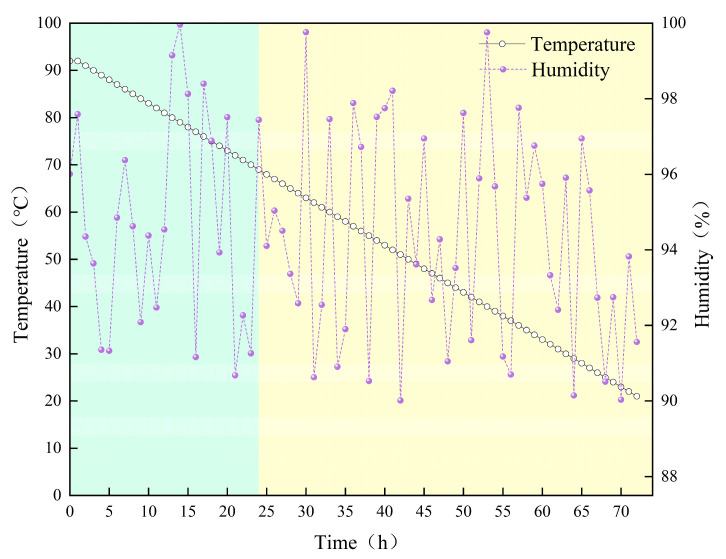
Variable-temperature curing profile.

To investigate the effects of curing age, temperature, and fiber content on the mechanical performance of concrete, six test conditions were implemented across three casting batches: Batch 1: Fiber-free concrete cured at constant temperature. Six specimens were cast and cured at 20 ± 1 °C with 95 ± 5% relative humidity for 72 h. After 24 h, the end surfaces of the specimens were ground flat and smooth. They were then divided into two groups: three specimens underwent immediate uniaxial compression testing, while the other three continued curing before testing. Batch 2: Fiber-free concrete subjected to variable-temperature curing. Six specimens were cured from 92 °C to 20 °C over 72 h, with a controlled temperature decrease of 1 °C per hour. After 24 h of curing, the end surfaces of the specimens were ground flat and smooth. Subsequently, three specimens underwent immediate uniaxial compression testing, while the other three continued curing until reaching a total curing age of 72 h. The curing profile is illustrated in Figure 2. Batch 3: Steel fiber-reinforced concrete specimens subjected to identical variable-temperature curing conditions and end-surface grinding methodology as Batch 2. Post-loading, all specimens were CT scanned to reconstruct their internal crack structures.

**Figure 3:**
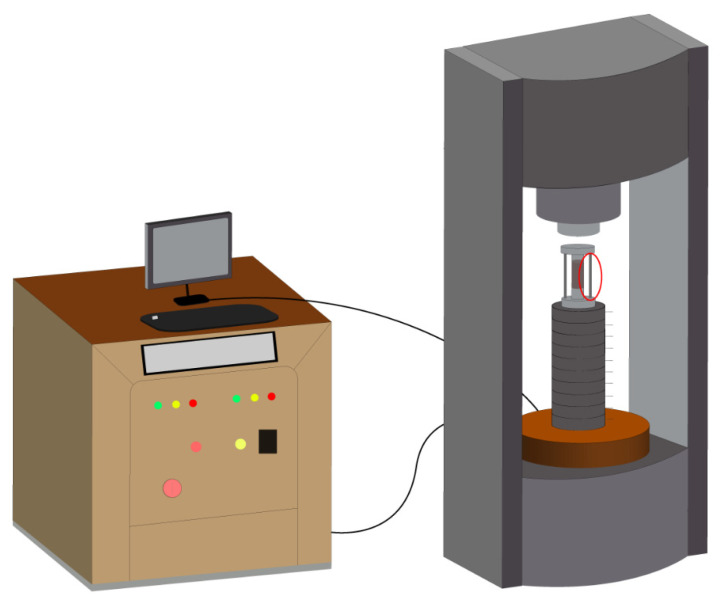
RST-416 uniaxial loading configuration (schematic).

Uniaxial loading was conducted using the apparatus shown in Figure 3. A built-in mechanical limit rod (circle in red) was used to prevent actuator overrun during specimen failure, thereby protecting sensitive components such as the force and displacement sensors. The specimens were loaded under displacement control at a rate of 0.1 mm/min.

After the loading was completed, the specimens were subjected to CT scanning, which can obtain the 3D crack characteristics and 2D CT slices of the internal structure of the specimens. The slices were processed through the machine learning module of ImageJ software to obtain the fractal characteristics of the cracks in the slices. ImageJ is a powerful open-source image processing software platform based on Java, developed by the National Institutes of Health (NIH), and is widely used in scientific research. The Random Forest workflow includes three stages: a training set, multiple decision trees, and an aggregated output via majority voting. In ImageJ, this is implemented through the following steps: (1) Initial Training: Crack features are labeled on several CT slices and designated as target classes. (2) Iterative Refinement: Initial classification results are manually corrected and reintroduced into the model. (3) Convergence: Repeated iterations effectively separate cracks from pores, even when the grayscale intensities are similar.

Finally, a functional model for the concrete specimens in the VT group and F group was initially constructed using Weibull statistical damage theory. This initially built functional model was used to fit the stress–strain curves of the VT group and F group obtained from the experiments. Through multiple iterative fittings, relevant model parameters can be obtained. When the functional model can no longer be iterated and the correlation coefficient R^2^ is relatively close to 1, it can be considered that the functional model at this point is the constitutive model for the specimens in the VT group and F group.

(1) $$\left\{\begin{array}{c} \sigma =\alpha {\left(\frac{\varepsilon }{\varepsilon_{A}}\right)}^{2}\sigma_{A}+\beta \frac{\varepsilon }{\varepsilon_{A}}\sigma_{A}\;\left(0\leq \varepsilon \leq \varepsilon_{A}\right)\\ \sigma =E_{C}\varepsilon_{C}\exp\left[-{\left(\frac{\varepsilon_{C}}{\lambda }\right)}^{k}\right]\;\left(\varepsilon_{A}\leq \varepsilon \leq \varepsilon_{P}\right) \end{array}\right.$$

## 3. Results

### 3.1. Mechanical Parameters

This study employed two curing ages for three types of concrete specimens: non-fiber specimens cured at room temperature (RT group), non-fiber specimens cured under variable temperature (VT group), and specimens incorporating 1% fiber cured under variable temperature (F group). This design resulted in six test combinations, with three parallel specimens prepared per combination for mechanical property testing.

**Figure 4:**
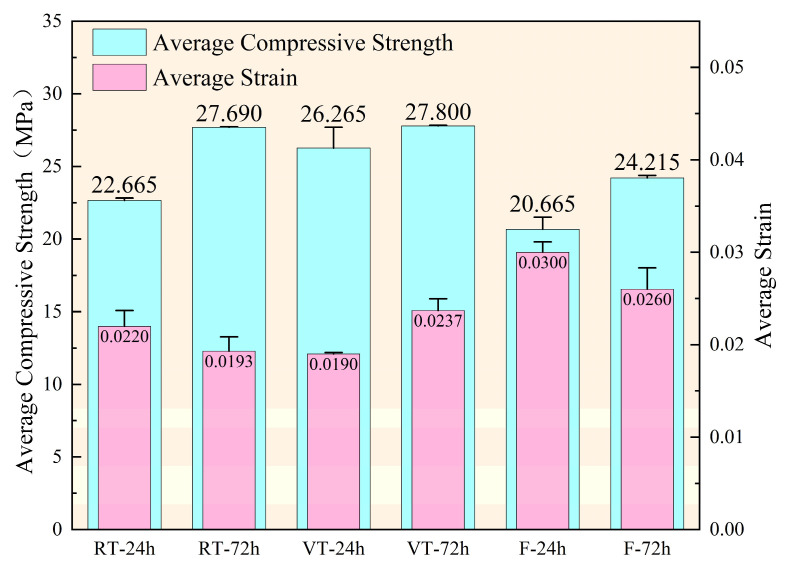
Mechanical test results of C30 concrete.

Outliers were identified and excluded using the following criterion: if the compressive strength or peak strain of a specimen deviated by more than 15% from the group mean, the data were considered statistically inconsistent and excluded. The arithmetic mean of the remaining two valid measurements was used for analysis. The uniaxial compressive strength and corresponding peak strain data are summarized in Figure 4.

The results indicate a positive correlation between compressive strength and hydration age across all curing conditions. However, the evolution of peak strain exhibited distinct trends among groups. In the RT and F groups, the peak strain decreased by 12.27% and 13.33%, respectively, with increasing age, indicating an age-related enhancement in stiffness. In contrast, the VT group showed a 24.74% increase in peak strain, suggesting that variable-temperature curing without fiber reinforcement induced thermal damage. This phenomenon implies that high-temperature curing accelerates microcrack development and weakens the interfacial transition zones, thereby compromising the mechanical integrity of concrete and potentially reducing its long-term structural durability.

### 3.2. Deformation Behavior

#### 3.2.1. Stress–Strain Curve Characteristics

**Figure 5:**
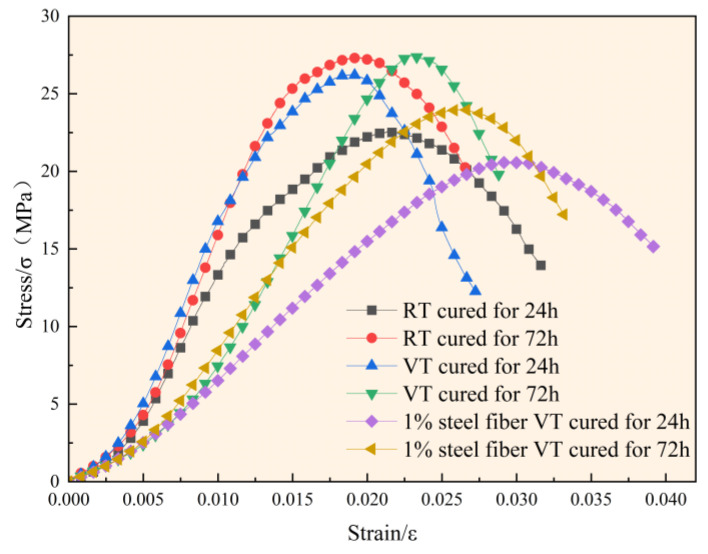
Mean stress–strain curves for each experimental condition.

The stress–strain curves obtained from the uniaxial compression tests are presented in Figure 5. As shown in Figure 5, the stress–strain responses of all specimens follow a typical four-stage pattern: compaction, elastic deformation, plastic deformation, and failure. The duration of each stage varies depending on the curing conditions and fiber content. The F group exhibited the longest compaction stage, primarily due to the incorporation of steel fibers, which increased internal air entrapment and micro-void formation [23]. As a result, greater initial deformation occurred before significant stress buildup. The elastic and plastic deformation stages were found to evolve with hydration age. Under identical curing conditions, advancing hydration prolonged the elastic stage and made the transition to the plastic stage more gradual. In the failure stage, the VT group showed a rapid decay of residual stress, while the F group exhibited a significantly slower decay. This contrast can be attributed to two mechanisms: first, high-temperature curing accelerated hydration, resulting in a denser but more brittle matrix; second, the presence of steel fibers in the F group effectively inhibited crack propagation, improving ductility and post-peak behavior. Furthermore, variations in the elastic modulus among groups are evident from the stress–strain curves. Both the RT and F groups showed an increased elastic modulus with hydration age. Conversely, the VT group exhibited a decrease in elastic modulus over time, along with an extended compaction stage in the 72-h specimens. This behavior can be explained by the accelerated hydration of C_3_S and C_3_A during the early high-temperature curing phase (0–24 h), which led to premature densification of the C-S-H gel. Although this initially increased the stiffness, it simultaneously inhibited subsequent hydration. Moreover, thermal expansion mismatch between aggregates (manufactured sand and crushed stone) and the cement matrix introduced interfacial shear stresses that exceeded the bonding strength [24]. These stresses induced microcracking and increased porosity in the 72-h specimens, ultimately extending the compaction phase.

#### 3.2.2. Compressive Strength and Elastic Modulus

The elastic modulus was determined as the slope of the linear (elastic) portion of the stress–strain curve. As shown in Figure 5, each of the six experimental conditions exhibited a relatively stable linear region following the end of the compaction stage, from which the elastic modulus was calculated. For the 24-h-aged specimens of the RT and VT groups, the compaction stage concluded at a strain of approximately 0.005. Therefore, the elastic modulus was evaluated over the strain interval from 0.005 to 0.01. The resulting values were 1.884 GPa, 2.324 GPa, and 2.348 GPa, respectively. For the remaining three specimens—characterized by a longer compaction stage—the modulus was calculated over the range of 0.01 to 0.02 strain. The obtained values were 1.720 GPa, 0.898 GPa, and 1.202 GPa, in the order tested.

**Figure 6:**
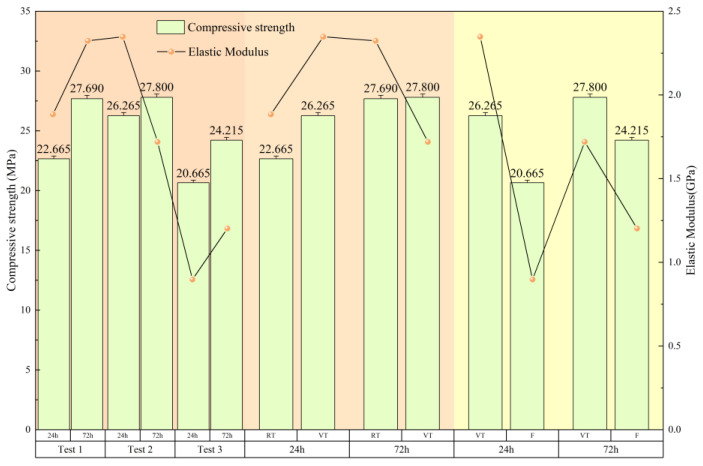
Compressive strength and elastic modulus. “Test 1,” “Test 2,” and “Test 3” refer to the RT, VT, and F groups, respectively.

Analysis of the test data in Figure 6 reveals that both the curing regime and fiber incorporation have significant impacts on the mechanical performance of concrete. On the left side of the figure, it is evident that that the compressive strength increased with curing age across all groups. In the RT group, the compressive strength increased by 22.17% from 24 to 72 h, accompanied by a 23.25% increase in elastic modulus. This reflects the beneficial effect of continued hydration at ambient temperature, which promotes matrix densification. In the VT group, while the 24-h compressive strength reached 26.265 MPa, the increase at 72 h was only 5.84%. More notably, the elastic modulus decreased by 26.75%, indicating that the high-temperature variable curing environment facilitates early hydration but also promotes the formation of microcracks and porosity, which limit long-term strength development.

In contrast, the F group (with added steel fiber content under variable-temperature curing) exhibited a 17.18% increase in compressive strength and a 33.85% increase in elastic modulus at 72 h. These results suggest that steel fibers mitigate the adverse effects of thermal damage by bridging microcracks and reinforcing the interfacial transition zone (ITZ) [25,26]. However, it is noteworthy that at 24 h, the F group displayed the lowest compressive strength (20.665 MPa) among the three, indicating that fiber incorporation may reduce early-age strength due to disrupted cement hydration and increased void content.

The central portion of Figure 5 highlights the influence of high-temperature variable curing alone. The 24-h compressive strength increased by 15.88% and the elastic modulus rose by 24.63% compared to ambient curing, confirming the accelerating effect of elevated temperature on early hydration. However, by 72 h, the strength had only marginally increased by 0.40%, while the elastic modulus declined by 25.99%. This deterioration in stiffness was accompanied by a noticeable increase in peak strain, which may adversely affect the structural rigidity and long-term durability of concrete.

The right-hand portion of the figure illustrates the influence of fiber addition under variable-temperature curing. Although the compressive strength and elastic modulus of the F group were lower than those of the VT group at an early age, they exhibited a more favorable development trend over time. Both properties increased with age, suggesting that fibers play a role in improving the mechanical properties of concrete in the later stages of curing.

In summary, ambient-temperature curing is more conducive to the progressive development of concrete strength. High-temperature variable curing, while beneficial for early-age strength, has a detrimental effect on long-term mechanical performance due to thermally induced cracking and interfacial weakening. However, the incorporation of steel fibers can effectively counteract these effects by limiting crack propagation and enhancing the ITZ, thus partially restoring mechanical integrity under harsh curing conditions.

### 3.3. Analysis of Energy Characteristics

**Figure 7:**
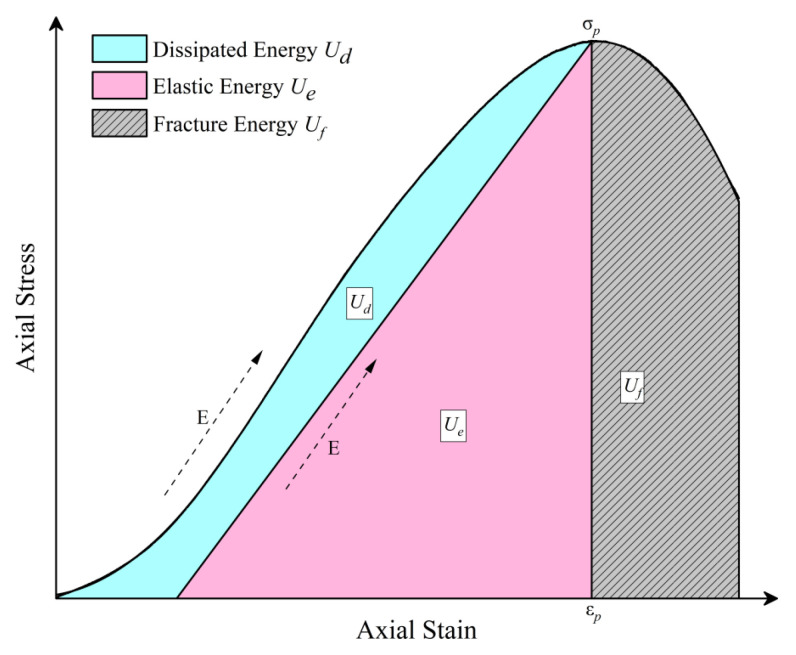
Energy evolution during the deformation process.

Energy evolution plays a critical role in characterizing the mechanical behavior of concrete. As illustrated in Figure 7, the stress–strain response of concrete can be decomposed into three principal energy components: dissipated energy ($U_{d}$), elastic energy ($U_{e}$), and fracture energy ($U_{f}$). These components are quantified using the following equations:

(2) $$U_{d}=\int \sigma d\varepsilon =U_{d}+U_{e}\left(\varepsilon \leq \varepsilon_{p}\right)$$

(3) $$U_{e}=\frac{\sigma \varepsilon }{2}=\frac{\sigma_{p}^{2}}{2E}$$

(4) $$U_{f}=\int \sigma d\varepsilon \left(\varepsilon \geq \varepsilon_{p}\right)$$

where $U_{d}$ represents the total energy absorbed by the specimen during loading, interpreted as the maximum energy stored before failure. *E* denotes the elastic modulus, *σ_p_* is the peak stress, and *ε_p_* is the peak strain.

**Figure 8:**
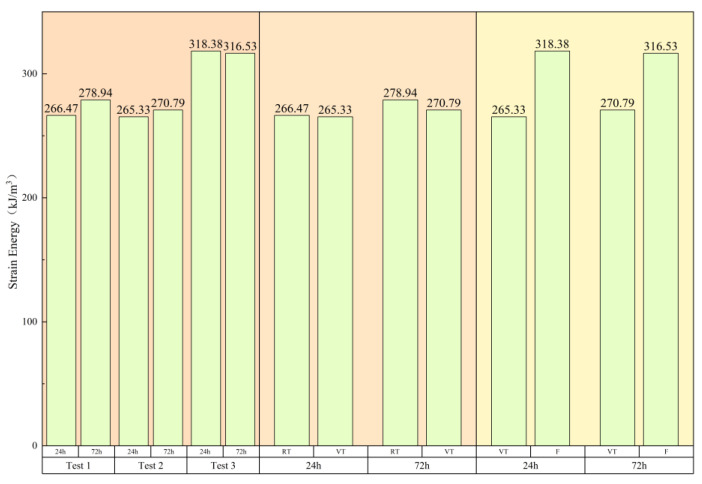
Energy evolution in concrete under different curing and reinforcement conditions.

In this study, only the absorbed energy was considered, reflecting the overall load-bearing capacity of the concrete. Based on Equation (2), the absorbed energy for the six averaged stress–strain curves was calculated as 266.47 kJ/m^3^, 278.94 kJ/m^3^, 265.33 kJ/m^3^, 270.79 kJ/m^3^, 318.38 kJ/m^3^, and 316.53 kJ/m^3^, respectively. The results are illustrated in Figure 8.

The left panel of Figure 8 presents the influence of hydration age on the three test groups. The RT group exhibited a 4.58% increase in energy absorption from 24 to 72 h, while the VT and F groups showed changes of 2.06% and −0.58%, respectively. These observations indicate the following:

RT Group: Under constant-temperature curing, gradual hydration improves matrix densification, enhancing the energy storage capacity with age.

VT Group: Although high-temperature curing accelerates early hydration, the process stabilizes by 72 h, resulting in minimal variation in absorbed energy.

F Group: The incorporation of steel fibers significantly enhances energy absorption, effectively counteracting the aging effect.

The central and right panels of Figure 8 isolate the effects of temperature and fiber addition, respectively. The central panel reveals a deterioration in energy absorption capacity in the VT group over time, with degradation increasing from 0.43% to 2.92%. This confirms that high-temperature variable curing compromises the material’s integrity, increasing fragility. The right panel shows that fiber reinforcement improves energy absorption by 20.00% at 24 h and 16.89% at 72 h. However, the relatively stable performance of the F group with age, compared to the slight improvements in the VT group, suggests that the relative efficacy of fiber reinforcement in mitigating thermal damage diminishes over time under high-temperature curing conditions.

### 3.4. Failure Patterns

#### 3.4.1. CT Slice Processing via Machine Learning

CT scanning is widely used for meso-scale investigations of concrete. In this study, micro-CT scanning was performed on damaged concrete specimens to assess their internal crack morphology. The CT system parameters were configured as follows: X-ray tube voltage of 200 kV, current of 180 μA, exposure time of 0.58 s, and frame count of 1080. The resulting 3D reconstructions were generated using Voxel Studio Recon software (2020), while Dragonfly was utilized to extract and export meso-scale crack distributions as 2D slice images.

Due to the relatively low resolution of CT imaging, initial noise reduction was applied prior to threshold-based segmentation. However, cracks and pores often share similar grayscale values in the CT slices, rendering conventional thresholding ineffective for accurate crack extraction.

To address this, the Random Forest algorithm—implemented via the Trainable Weka Segmentation plugin in ImageJ—was employed. ImageJ is well known for its advanced plugin architecture, precise measurement tools, and automation capabilities [27,28]. The Random Forest algorithm, an ensemble learning method that aggregates decisions from multiple classification trees, is widely applied in biomedical image analysis [29]. While it may exhibit occasional errors in segmenting microcracks, it performs well in distinguishing macroscopic crack features typical of concrete subjected to uniaxial compression.

In this study, cracks in post-failure concrete specimens were predominantly macroscopic and, thus, suitable for detection at micron-level resolution. The Random Forest algorithm enabled effective segmentation of cracks even under low-resolution conditions.

**Figure 9:**
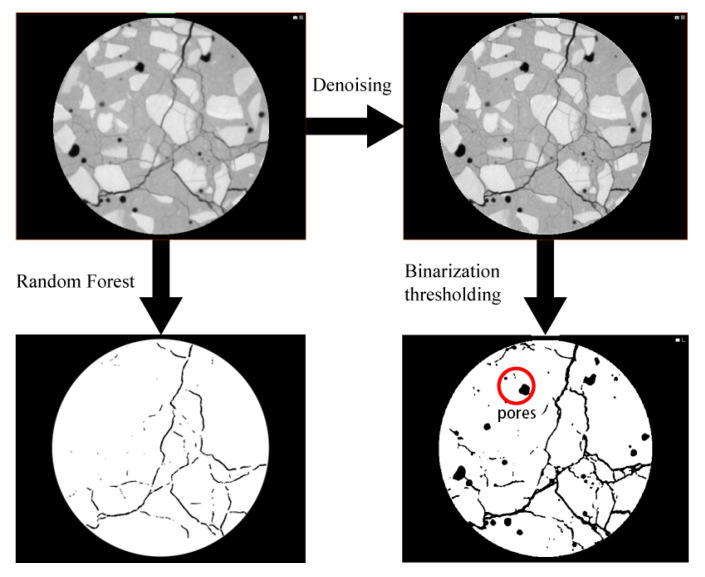
Comparison between traditional thresholding and Random Forest-based crack segmentation.

The dataset was divided into training and testing sets at a ratio of 80:20. As shown in Figure 9, the Random Forest-based segmentation significantly outperformed traditional binary thresholding. The thresholding method failed to distinguish cracks from pores, and smaller cracks were often misclassified due to grayscale overlap with the matrix. In contrast, the machine learning approach accurately extracted both primary and secondary crack networks, demonstrating its suitability for meso-scale damage analysis of concrete.

#### 3.4.2. Machine Learning-Based CT Image Analysis

**Figure 10:**
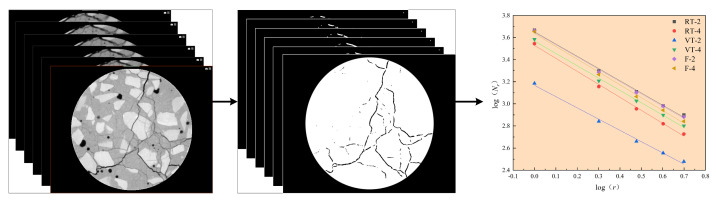
Workflow for calculating the fractal (box-counting) dimensions of cracks in CT images.

Figure 10 illustrates the workflow for determining the box-counting dimension of cracks in CT slice images. Six representative concrete specimens (one from each of three experimental groups at two different hydration ages) were selected, with each specimen comprising 807 CT slices. CT slices from identical spatial positions across all specimens were randomly extracted and processed using the trained Random Forest model to generate binary images isolating cracks.

Based on fractal theory [30], the box-counting method was applied to these binary images to quantify the fractal dimensions of the crack networks. In this study, the 400th slice from each specimen was randomly selected for initial analysis. The calculated box-counting dimension values and corresponding fitting equations are presented in Table 2. The log–log plots of Nr versus r displayed strong linearity, with correlation coefficients (R^2^) approaching 1.0, confirming a robust fractal relationship between crack area and measurement scale. These findings demonstrate that cracks in concrete exhibit clear fractal characteristics.

**Figure 11:**
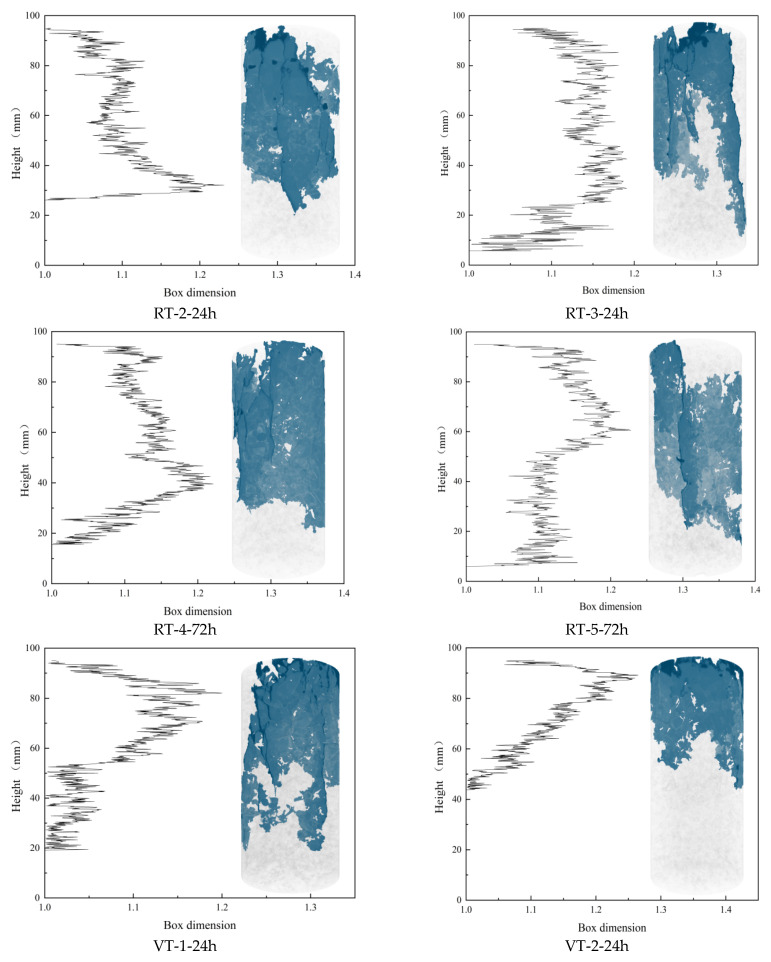

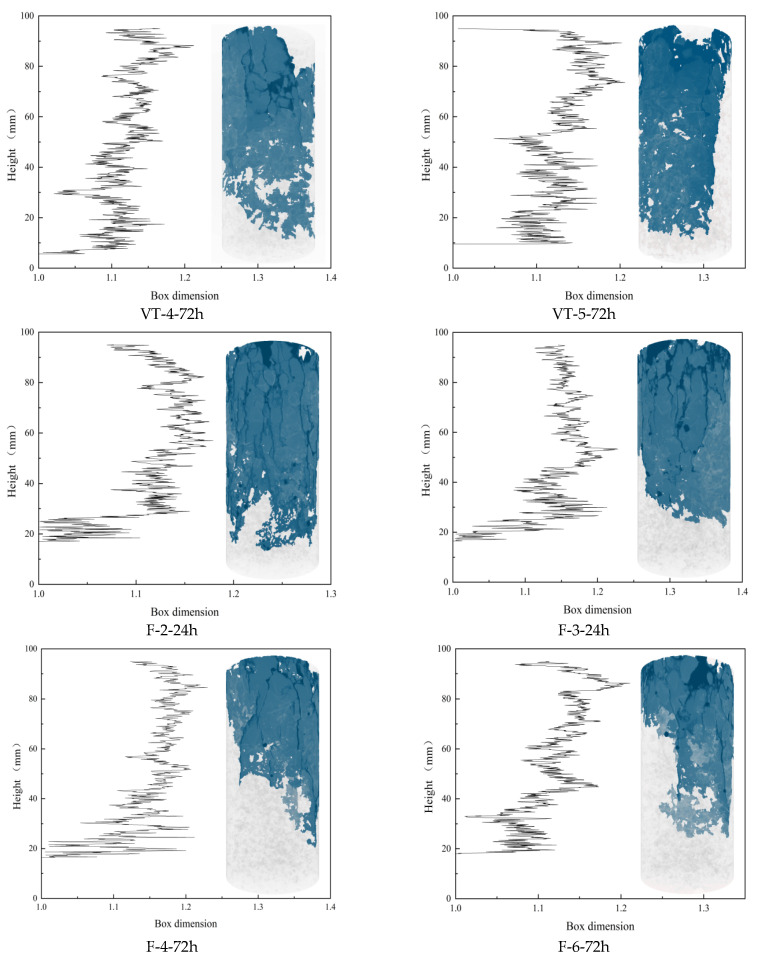
Distribution of box-counting dimensions along specimen height for different experimental conditions. In the figure, the black polyline on the left represents the box-counting dimension characteristics along the height direction of the specimen, while the right side shows the 3D CT reconstruction image of the corresponding specimen, with the blue area indicating the internal failure cracks of the specimen. Regarding the specimen naming convention (e.g., RT-1-24h): RT = curing condition (room temperature); 1 = specimen identification number; 24h = curing duration (24 h).

To further validate the analysis, the box-counting dimensions of all 807 slices from the six specimens were calculated. The results are shown in Figure 11, which reveals that the distribution of fractal dimensions along the specimen height aligns well with the 3D crack distribution observed in the CT reconstructions. This strong agreement supports the reliability of the Random Forest algorithm in extracting crack features.

**Figure 12:**
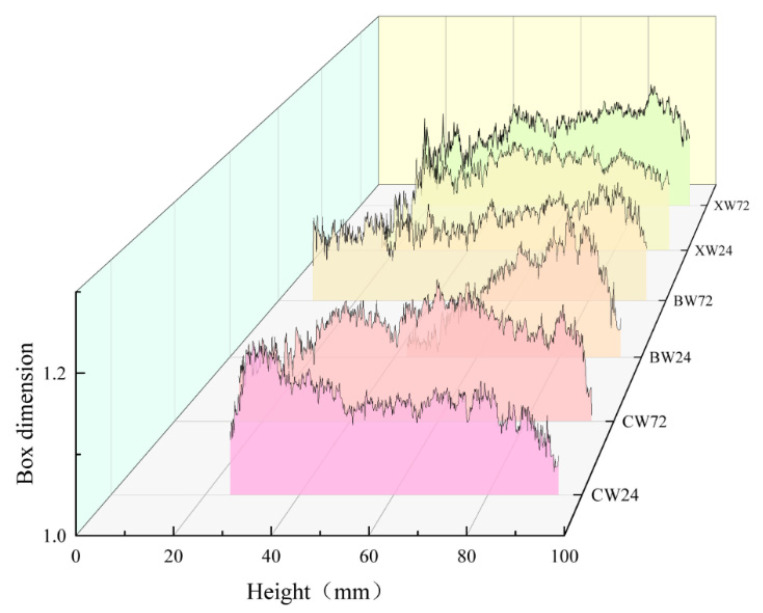
Average box-counting dimension of cracks along specimen height for each experimental group.

Figure 11 also highlights the non-uniform spatial distribution of cracks. Due to axial loading from the top surface, cracks predominantly developed in the upper regions of the specimens. To further investigate the evolution of fractal characteristics, the box-counting dimensions of slices from specimens under identical curing conditions were averaged, with the results presented in Figure 12. A general trend emerges: the fractal dimension increases initially, stabilizes across the mid-height range, and then decreases toward the base.

Notably, the trends differ between the VT and RT groups at 24 h: In the VT group (24 h), the fractal dimension decreases to 1 at approximately 42 mm height. In the RT group (24 h), the fractal dimension increases near 30 mm height.

Mechanistic Interpretation: VT Group: High-temperature curing accelerates hydration, producing abundant but spatially heterogeneous hydration products. This causes local variations in strength and energy absorption. As a result, the energy transfer paths deviate from the loading axis and propagate toward weaker zones. Crack growth was hindered in the middle of the specimen and then propagated to the surrounding weak areas, resulting in premature failure of the specimen. RT Group: In contrast, slower hydration results in insufficient formation of hydration products, creating weak zones. Energy tends to concentrate in these regions under loading, increasing the crack density and raising the box-counting dimension.

Except for two anomalous curves, most of the profiles in Figure 12 follow a three-phase trend: initial increase, stabilization, and final decline. This can be explained as follows: (1) Initial Phase: At the onset of loading, the interface between the specimen and the loading apparatus does not store significant energy. Instead, elastic energy accumulates around the 85 mm height mark. Once a critical energy threshold is reached, microcracks initiate and rapidly propagate, leading to high local fractal dimensions. (2) Stabilization Phase (35–80 mm): In this region, damage intensifies as loading continues. The RT group exhibits the highest box-counting dimensions, followed by the F and VT groups. This indicates that slow hydration in the RT group leads to greater internal damage. The F and VT groups, cured at elevated temperatures, form more hydration products, which enhance their resistance to cracking. However, the presence of fibers in the F group introduces additional porosity, increasing the damage slightly compared to the VT group. (3) Final Decline Phase: Toward the lower sections of the specimen, fewer cracks are observed, indicating reduced damage. This reduction is most pronounced in the F group, where fiber bridging strengthens the matrix and improves interfacial bonding. As a result, the F specimens retain larger undamaged areas, highlighting the beneficial role of fiber reinforcement in mitigating thermal damage during variable high-temperature curing.

## 4. Discussion

### 4.1. Constitutive Model Development

Under uniaxial compression, the damage behavior of concrete can be conceptualized as a combination of damaged and undamaged regions. Based on Lemaitre’s damage mechanics theory, the constitutive relationship of concrete under uniaxial compression can be expressed as follows:

(5) $$\sigma =E\varepsilon \left(1-D\right)$$

where *D* represents the damage variable of the concrete material.

For the VT group specimens, the total damage process under uniaxial loading can be divided into two stages: thermal damage induced by high-temperature variable curing, and loading-induced damage under external mechanical stress.

The thermal damage variable resulting from the first stage is defined as follows:

(6) $$D_{B}=1-\frac{E_{B}}{E}$$

where $E_{B}$ represents the elastic modulus of VT group concrete, and E is the elastic modulus of RT group concrete. Thus, *D_B_* represents the damage due to high-temperature variable curing conditions.

In the second stage, the material’s internal structure—characterized by micro-voids and -cracks—undergoes progressive damage due to continued external loading. Assuming that the strength of mesoscopic damaged elements follows a Weibull distribution, the probability density function is given by

(7) $$F\left(x\right)=\frac{k}{\lambda }{\left(\frac{x}{\lambda }\right)}^{k-1}\exp\left[-{\left(\frac{x}{\lambda }\right)}^{k}\right]$$

where *x* represents the strength of a damaged meso-element, and *k* and *λ* are the Weibull parameters.

The number of failed meso-elements (Ns) within the interval [0, x] is

(8) $$N_{S}=\int_{0}^{x}NF\left(x\right)dx$$

where *N* represents the total number of meso-elements. Consequently, the second-stage damage variable is

(9) $$D_{L}=\frac{N_{S}}{N}=\int_{0}^{x}F\left(x\right)dx=1-\exp\left[-{\left(\frac{x}{\lambda }\right)}^{k}\right]$$

Assuming that failure follows the maximum tensile strain criterion, the strength variable can be expressed as follows [31]:

(10) $$x=x\left(\varepsilon \right)=\varepsilon_{B}$$

where *ε_B_* represents the strain of VT group concrete.

Substituting Equation (10) into Equation (9) and incorporating thermal damage from the first stage yields the total damage variable:

(11) $$D=1-\frac{E_{B}}{E}\exp\left[-{\left(\frac{x}{\lambda }\right)}^{k}\right]=1-\frac{E_{B}}{E}\exp\left[-{\left(\frac{\varepsilon_{B}}{\lambda }\right)}^{k}\right]$$

Substituting Equation (11) into the constitutive relationship (Equation (5)) gives the statistical constitutive model for the VT group:

(12) $$\sigma =E_{B}\varepsilon_{B}\exp\left[-{\left(\frac{\varepsilon_{B}}{\lambda }\right)}^{k}\right]$$

Similarly, the constitutive model for the F group is expressed as follows:

(13) $$\sigma =E_{X}\varepsilon_{X}\exp\left[-{\left(\frac{\varepsilon_{B}}{\lambda }\right)}^{k}\right]$$

where *E_X_* and *ε_X_* represent the elastic modulus and strain of the F group concrete, respectively.

To account for the compaction stage prior to strain hardening, Lu et al. [32] proposed the following expression:

(14) $$\sigma =\sigma_{A}{\left(\frac{\varepsilon }{\varepsilon_{A}}\right)}^{2}$$

where *σ_A_* and *ε_A_* represent the maximum stress and strain during the compaction and closure of initial voids and microcracks.

However, directly adopting Equation (14) is not suitable for accurately modeling this stage. To address this, the correction factors α and β are introduced, and the modified expression becomes

(15) $$\sigma =\alpha {\left(\frac{\varepsilon }{\varepsilon_{A}}\right)}^{2}\sigma_{A}+\beta \frac{\varepsilon }{\varepsilon_{A}}\sigma_{A}$$

For this model to remain valid at the transition point (*ε* = *ε_A_*, *σ* = *σ_A_*), it must satisfy the condition *α* + *β* = 1. Considering the compaction stage, the constitutive models for the VT and F groups can be further refined as follows:

(16) $$\sigma =E_{B}{(\varepsilon }_{B}-\varepsilon_{A})\exp\left[-{\left(\frac{\varepsilon_{B}-\varepsilon_{A}}{\lambda }\right)}^{k}\right]$$

(17) $$\sigma =E_{X}{(\varepsilon }_{X}-\varepsilon_{A})\exp\left[-{\left(\frac{\varepsilon_{X}-\varepsilon_{A}}{\lambda }\right)}^{k}\right]$$

Combining both the compaction and strain-hardening stages, the complete constitutive model for concrete is expressed as follows:

(18) $$\left\{\begin{array}{c} \sigma =\alpha {\left(\frac{\varepsilon }{\varepsilon_{A}}\right)}^{2}\sigma_{A}+\beta \frac{\varepsilon }{\varepsilon_{A}}\sigma_{A}\;\left(0\leq \varepsilon \leq \varepsilon_{A}\right)\\ \sigma =E_{C}\varepsilon_{C}\exp\left[-{\left(\frac{\varepsilon_{C}}{\lambda }\right)}^{k}\right]\;\left(\varepsilon_{A}\leq \varepsilon \leq \varepsilon_{P}\right) \end{array}\right.$$

where *E_C_* represents the elastic modulus after compaction, and $\varepsilon_{C}$ represents the strain offset following the compaction phase.

Finally, based on geometric constraints derived from the average stress–strain curve, the Weibull parameters *k* and *λ* can be determined using

(19) $$\left\{\begin{array}{c} k={\left[\ln\left(\frac{E_{C}∆\varepsilon }{∆\sigma }\right)\right]}^{-1}\\ \lambda =∆\varepsilon k^{\frac{1}{k}} \end{array}\right.$$

where *Δε* and *Δσ* represent the differences between the peak and compaction stage values for strain and stress, respectively.

### 4.2. Verification of the Constitutive Model

**Figure 13:**
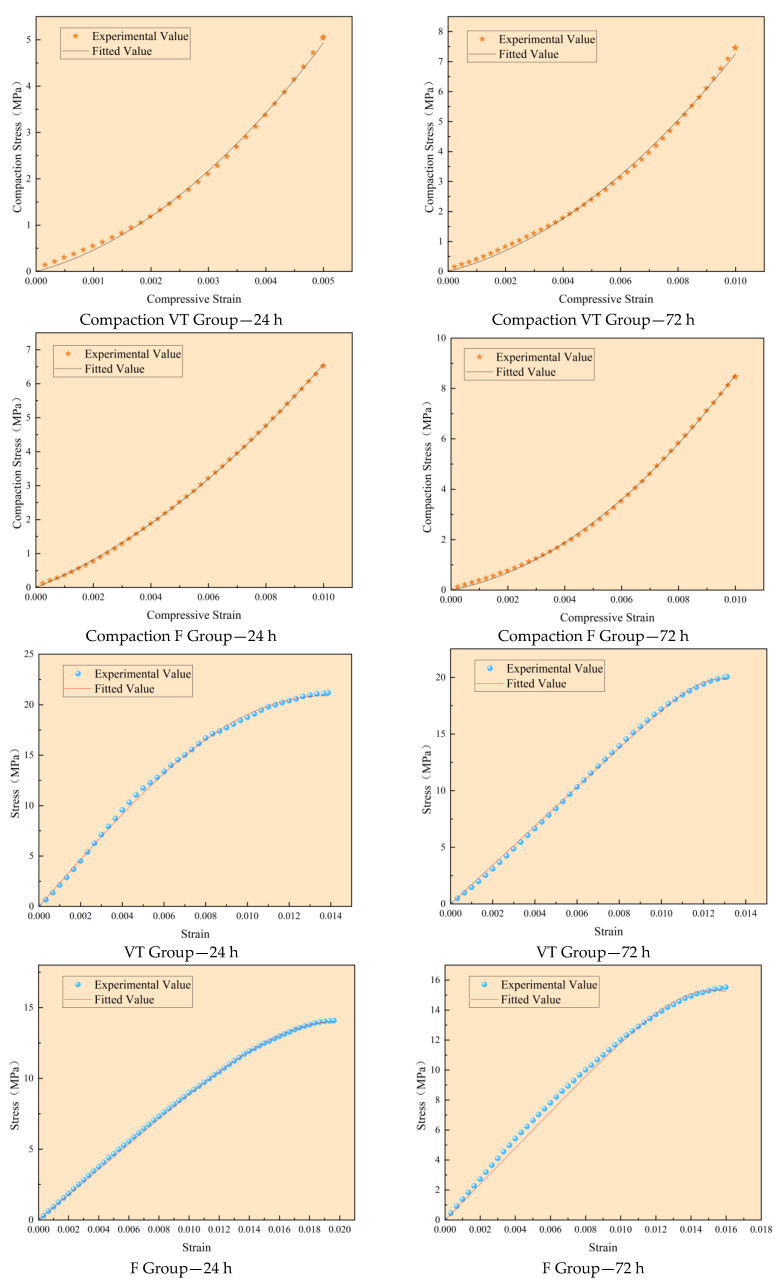
Comparison of experimental and theoretical stress–strain curves.

Based on the uniaxial compression test results, the parameters of the proposed damage constitutive models were determined using the average stress–strain curves for the VT and F concrete groups. The calculated parameters are presented in Table 3, and the corresponding model fitting results are shown in Figure 13.

The values of α + β for the four datasets are 0.9734, 0.9713, 1.0068, and 1.0069, all of which closely approximate to 1.0. This satisfies the necessary condition of Equation (15), confirming the validity of the modified compaction-stage model. Additionally, the correlation coefficient R^2^ for both the compaction and elastic–plastic stages exceeds 99%, indicating excellent agreement between the experimental and theoretical results.

Notably, the F group exhibits values of α + β closer to 1.0 and slightly higher R^2^ values than the VT group, suggesting that the proposed constitutive model better captures the mechanical behavior of steel fiber-reinforced concrete under high-temperature variable curing conditions. However, the model demonstrates limited capability in describing post-peak softening behavior.

In the field of compressive constitutive models for concrete specimens, the academic community has achieved fruitful research results. At present, the research hotspots of concrete constitutive models mainly focus on the “plasticity–damage coupling mechanism” of concrete, the “compressive constitutive model with automatic recovery of tension-compression stiffness” [17], the compressive constitutive model of fiber-reinforced concrete [33], the compressive constitutive model of fiber-reinforced concrete under high-temperature environments [34], and other aspects. However, there are relatively few studies on constitutive models of early-age steel fiber-reinforced concrete under the variable-temperature environment of high-ground-temperature tunnels. In this study, a constitutive model of steel fiber-reinforced concrete under variable-temperature environments was constructed by combining Weibull statistical damage theory with Lemaitre damage mechanics, so as to quantify the temperature-induced damage to early-age concrete under the characteristic “spraying–heating–cooling” temperature change process of high-ground-temperature tunnels. By introducing compaction stage correction coefficients (α, β), a multi-stage constitutive equation is established, which can simultaneously characterize pore compaction, elastic deformation, and damage evolution. Experimental verification shows that the model has high accuracy (goodness-of-fit R^2^ > 99%) and especially solves the problem that traditional models cannot describe the interface deterioration under high temperatures. This model provides a key theoretical tool for the numerical simulation of support structures, optimization of fiber content, and design of construction and maintenance processes in high-ground-temperature tunnels, thereby directly improving the safety control level and durability.

### 4.3. Discussion on Early-Age Concrete Strength in High Geothermal Tunnels

According to relevant regulations [35], the strength grade of concrete used in underground engineering construction should not be lower than C25, and the 1-day compressive strength should not be less than 8 MPa. The concrete used in this study was of strength grade C30, and the 1-day compressive strength of all three test groups exceeded 20 MPa, which meets the requirements of tunnel construction specifications.

Further analysis based on the test results in Section 3.1 shows that the strength of specimens in the RT group increased by 22.17% with age, while that in the F group increased by 17.18%, and that in the VT group only increased by 5.84%. Although the strength of all three groups of specimens increased with age, the growth rate of the VT group lagged significantly, and its elastic modulus showed a downward trend. This phenomenon indicates that the high-temperature and high-humidity environment can significantly accelerate the hydration process of concrete within 0–24 h. However, the early rapid hydration caused by it will reduce the uniformity of the internal structure of the specimens and increase the strength difference in various regions, which, in turn, leads to the weak growth of later strength and more serious damage when the specimens are destroyed.

It is worth noting that the compressive strength of the F group at 1d and 3d ages is slightly lower than that of the RT group. This is mainly due to the weakening of the interfacial bonding performance between steel fibers and the concrete matrix under high-temperature conditions. High temperature causes the bonding gap between the fibers and cement mortar to increase, forming micro-porosity aggregation areas, thus having a certain negative impact on early strength. Nevertheless, the later strength development trend of the F group is significantly better than that of the VT group, and its advantages in resisting high-temperature damage (such as inhibiting crack propagation and improving toughness) are more prominent. Therefore, considering both the compliance of early strength and the stability of long-term performance, it is recommended to use a mix ratio with a fiber volume content greater than 1% for pouring in tunnel construction.

In addition, to further weaken the adverse impact of high temperatures on the fiber–matrix interfacial bonding performance, future research could explore the use of high-temperature-resistant fibers (such as basalt fibers and carbon fibers) or surface modification treatment of steel fibers (such as coating with high-temperature adhesives). By optimizing the structure of the interfacial transition zone between fibers and the matrix, the early strength can be ensured, and the inhibitory effect of fibers on high-temperature damage to concrete can be brought into full play, which will also be an important direction for subsequent research.

## 5. Conclusions

Concrete structures in high geothermal tunnel environments are often subjected to complex thermal–mechanical coupling effects during early curing, which significantly affect their hydration process, mechanical performance, and long-term durability. This study systematically investigated the influence of high-temperature variable curing on the early-age hydration, mechanical properties, and damage evolution of concrete, integrating experimental testing, image analysis, and theoretical modeling to provide comprehensive insights for engineering applications.

High-temperature variable curing significantly accelerates the early-age hydration of concrete, leading to a 15.88% increase in 1-day compressive strength compared to constant-temperature curing. However, this curing regime causes progressive degradation of mechanical properties as aging proceeds. Although the incorporation of steel fibers reduces the early compressive strength by 8.82% due to air entrainment, it effectively mitigates thermal damage induced by high-temperature variability, demonstrating its potential for improving concrete resilience in thermally challenging environments.

To address the challenges of crack characterization in damaged concrete, this study employed the Random Forest algorithm within ImageJ’s machine learning module to extract crack features from low-resolution CT slices of load-damaged specimens. The fractal dimension distributions along the height direction derived from this method showed high consistency with 3D-reconstructed crack morphologies, validating the effectiveness of this approach for reliable CT image analysis in concrete fracture studies.

Furthermore, a constitutive model for early-age concrete under high-temperature variable curing was developed by integrating Lemaitre’s damage mechanics theory with the Weibull statistical distribution. This model was successfully validated against experimental data, with a goodness-of-fit exceeding 99%, and accurately captured the complete stress–strain behavior of both plain and steel fiber-reinforced concrete under coupled thermal–mechanical loading.

Based on these findings, for concrete construction in high geothermal tunnels with elevated surrounding rock and groundwater temperatures, the use of steel fiber-reinforced concrete with a fiber dosage exceeding 1% by volume is strongly recommended. Additionally, controlling the cooling rate during curing is critical to balance the need for high early-age strength and long-term durability. These results not only deepen the understanding of concrete performance under high-temperature variable conditions but also provide practical guidance for enhancing the reliability and sustainability of concrete infrastructure in thermally harsh engineering environments.

## Figures and Tables

**Table 1:** Mix proportion of C30 concrete (kg/m^3^).

Material	Cement	Fine Aggregate	Coarse Aggregate	Water Reducer	Water	Steel Fiber	Accelerator
Dosage	472	912	777	4.72	166	35	4.72

**Table 2:** Fitting results of box-counting dimensions for representative CT slices.

Specimen Number	Fitting Equation	Box-Counting Dimension (*D*)	R^2^
RT-2	y=−1.1075x+3.6520	1.1075	0.9964
RT-4	y=−1.1758x+3.5293	1.1758	0.9972
VT-2	y=−1.0175x+3.1672	1.0175	0.9947
VT-4	y=−1.1166x+3.5681	1.1166	0.9971
F-2	y=−1.1123x+3.6454	1.1123	0.9968
F-4	y=−1.1595x+3.6360	1.1595	0.9967

**Table 3:** Model parameters for concrete.

Sample Number	Test Condition	α	β	*R* ^2^	k	λ	*R* ^2^
1	VT cured for 24	0.6439	0.3295	0.9980	2.2864	0.0198	0.9988
2	VT cured for 72	0.6327	0.3386	0.9979	9.9077	0.0161	0.9990
3	1% steel fiber VT cured for 24	0.4746	0.5322	0.9999	4.2575	0.0277	0.9993
4	1% steel fiber VT cured for 72	0.7485	0.2584	0.9994	5.6037	0.0207	0.9937

## Data Availability

The original contributions presented in this study are included in the article. Further inquiries can be directed to the corresponding author.

## Abbreviations

The following abbreviations are used in this manuscript:

RT	Room-temperature group
VT	Varying-temperature group
F	Fiber group
